# Effect of Donor Cigarette Smoking in Kidney Transplantation: Re-Evaluation of Long-Term Outcomes

**DOI:** 10.3389/ti.2024.12955

**Published:** 2024-06-24

**Authors:** Felix Becker, Nicola Sariye Pollmann, Ricarda Funke-Kaiser, Dennis Görlich, Shadi Katou, Haluk Morgül, Felicia Kneifel, Stefan Reuter, Andreas Pascher, Philipp Houben

**Affiliations:** ^1^Department of General, Visceral and Transplant Surgery, University Hospital Münster, Münster, Germany; ^2^Institute of Biostatistics and Clinical Research, University Hospital Münster, Münster, Germany; ^3^Department of Medicine D, Division of General Internal Medicine, Nephrology and Rheumatology, University Hospital Münster, Münster, Germany

**Keywords:** kidney transplantation, donor criteria, smoking, graft survival, patient survival

## Abstract

Cigarette smoking is a common risk factor associated with negative long-term outcomes in kidney transplant recipients. However, whether donor smoking decreases graft longevity or negatively impacts recipient survival after kidney transplantation remains unknown. Therefore, this study aims to investigate the long-term outcome in patients who received a kidney graft from a deceased smoking or non-smoking donor. A total of 580 patients were divided into two groups: patients who received a graft from a smoking donor (*n* = 276) and those who received a graft from a non-smoking donor (*n* = 304). Analysis of demographic factors showed that the non-smoking cohort was older, had more extended criteria donors and longer warm ischemia times. The primary composite endpoint of patient and graft survival was better in the smoking donor cohort when analyzed using Kaplan-Meier method but not when controlled for covariates in multivariate analyses. These findings do not support a previously reported negative impact of deceased donor smoking on kidney transplant recipients. Thus, the underlying results should not be interpreted in favor of a positive donor smoking history, but rather remind the transplant community that donor smoking should not be considered as a deciding factor in refusing an otherwise acceptable kidney graft.

## Graphical Abstract

**Figure:**
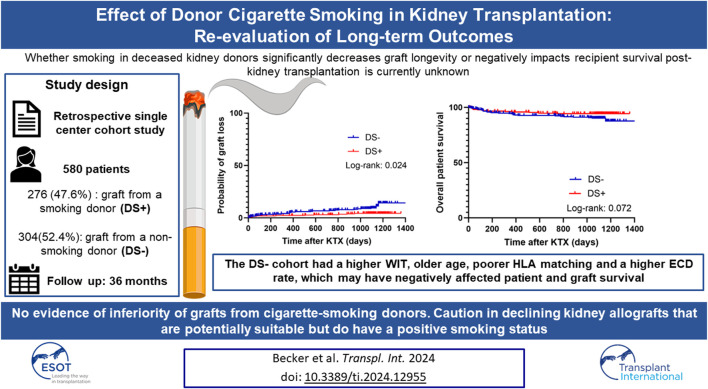
Created with BioRender.com.

## Introduction

Critical evaluation of donor-associated characteristics in kidney transplantation (KTX) represents an ever-growing topic since the ongoing paucity of kidney grafts remains a cardinal problem in transplant medicine. This demands optimal utilization of every potentially suitable organ. Nevertheless, a high percentage of kidney grafts is still discarded, and several donor-associated characteristics have been identified that contribute to this, including donor age, diabetes, hypertension, and death from cerebrovascular accidents [1, 2]. However, for other donor-associated characteristics, one faces the dilemma of a yet not fully elucidated impact on outcomes following KTX. This eventually results in discarding suitable kidney grafts, further contributing to the ever-growing organ shortage. Nevertheless, potentially harmful donor-associated characteristics pose a risk for impaired outcomes after KTX and should, therefore, be avoided [3]. Donor smoking (DS) is a common and thus highly relevant potential donor-associated risk factor that has only been poorly studied for its impact on long-term outcomes post-KTX.

The World Health Organization reports that 22.3% of the world’s population used tobacco in 2020, making it the leading risk factor for death among men [4, 5]. In particular, a high prevalence of smoking (15.7% in 2018) has been observed over the past 20 years among 55–64 olds, who represent the majority of today’s donor cohort [6]. There is a large body of evidence linking cigarette smoking in KTX recipients to multiple adverse events, including an increased likelihood of cardiovascular events, risk of death and graft loss [7]. A negative smoking history or smoking cessation, even after the start of renal replacement therapy, is highly beneficial, as a 5-year smoking cessation before KTX has been shown to reduce the risk of graft failure [8]. While cigarette smoking in KTX recipients impairs patient and graft survival and long-term functional outcomes [7, 9], data for kidney recipients who received a graft from a smoking donor is still limited. Only a few studies have investigated the impact of DS in KTX and have reported inconsistent results regarding graft and recipient survival [10–13]. Of interest, none of these studies were conducted within the Eurotransplant (ET) region, solely used brain-dead donors, or included patients from the last decade.

Although there is little evidence that the quality of kidney grafts from smoking donors is compromised, DS is among the factors that significantly increase the discard odds for kidney grafts [14]. One possible explanation is that smoking is associated with the development of glomerulosclerosis and the progression of pre-existing renal diseases. As recently confirmed by Ataka et al., the rate of glomerulosclerosis was increased in smoking living kidney donors [15]. Nevertheless, the significance of these pathological changes is still unclear for long-term outcomes after KTX, especially in deceased donors [14]. Additionally, smoking is associated with the development of arteriosclerosis, which could be a crucial factor in discarding organs from smoking donors, at least from a surgical point of view [16]. A high likelihood of arteriosclerosis represents a technical challenge and thus increases the risk of prolonged warm ischemia and early graft loss due to vascular complications.

Notably, whether smoking in deceased kidney donors significantly decreases graft longevity or negatively impacts recipient survival post-KTX remains unknown. Therefore, there is an unmet need for further investigation and the practice of discarding kidneys from cigarette-smoking donors, regardless of organ quality, should be critically re-evaluated. Hence, this study aims to investigate the effects of DS on long-term patient, graft, and functional outcomes post-KTX in a contemporary cohort from the ET region.

## Materials and Methods

### Study Design and Study Population

**FIGURE 1:**
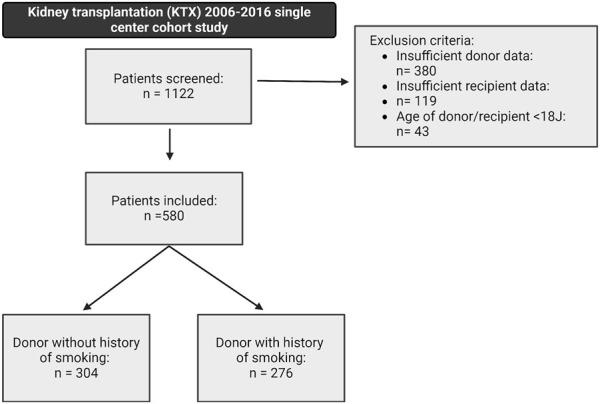
Study design and patient selection within the underlying retrospective cohort study, including a 36-month follow-up. A total of 580 patients met the following inclusion criteria: kidney transplantation after brain-dead donation, donor or recipient age above 18 years, and a complete donor and recipient dataset. Patients were stratified into two groups: (1) patients receiving a graft from a smoking donor (DS+) and (2) patients receiving a graft from a non-smoking donor (DS−).

The study design was a retrospective single-center cohort study with a 36 months follow-up. The initial study population comprised patients who received a kidney graft at the University Hospital Münster, Germany, between 2006 and 2016. Patients were screened for inclusion if they met the eligibility criteria of being over 18 years of age, transplanted with a post-mortem donated kidney, and without combined organ transplantation. A total of 1,122 patients were identified, of whom 542 were excluded due to insufficient donor or recipient data or not meeting the inclusion criteria (Figure 1). The remaining 580 patients met the eligibility criteria and were further stratified into two groups: 1) patients who received a graft from a smoking donor (DS+) and 2) patients who received a graft from a non-smoking donor (DS−). All the data used in the analysis were de-identified. Written informed consent was weaved because the study was a retrospective chart review. The study was conducted in accordance with the ethical principles in the Declaration of Helsinki. The local ethics committee approved the conduct of the study (Ethik-Kommission Westfalen-Lippe, permit number: 2021-788-f-S).

### Patient Cohort and Outcome Characteristics

Only kidney grafts from brain-dead donors were included in the study. All grafts were procured on behalf of ET, and donor characteristics were obtained from the Eurotransplant Network Information System (ENIS). Recipient data were collected retrospectively from a prospective clinical database. Donor characteristics included age, sex, body mass index (BMI), cardiopulmonary resuscitation (CPR), duration of CPR (in minutes), presence of hypertension or diabetes mellitus, cold and warm ischemia time (WIT), need for vasopressors during donor evaluation, length of stay in the intensive care unit prior to donation, highest and most recent (at time of procurement) serum creatinine (sCr) levels (in µmol/L) during donor evaluation, diuresis before donation, cytomegalovirus (CMV) status, human leukocyte antigen (HLA) mismatch, and presence of more than one renal artery. Additionally, the kidney donor risk index (KDRI) and kidney donor profile index (KDPI) were calculated using the known variables [17]. Extended criteria donor (ECD) status was defined as age ≥60 years or 50–59 years with at least two of the following conditions: a history of hypertension, a sCr level of 1.5 mg/dL, and a cerebrovascular cause of death. Recipient characteristics involved age, sex, dialysis vintage, history of hypertension, and the reason for end-stage kidney disease (ESKD).

### Outcome Parameters

A composite endpoint (event-free survival) was defined as the primary endpoint and included graft loss and patient survival. Graft loss was defined as the need to reinitiate dialysis. The primary endpoint was estimated using the Kaplan-Meier method and compared using the log-rank test.

The postoperative routine follow-up was conducted three (baseline), 6, 12, 24, and 36 months post-KTX. Blood and urine samples were collected immediately postoperatively and during routine follow-up. Renal function was defined as a secondary outcome parameter and was measured by the estimated glomerular filtration rate (eGFR; mL/h/1.73 kg^2^ and estimated using the Chronic Kidney Disease Epidemiology Collaboration [CKD EPI] formula), protein excretion (PE) per day (mg/d), and urine protein/creatinine ratio (UPCR, mg/g creatinine). Other secondary outcome measures included primary non-function (PNF, defined as the need for continued dialysis within 90 days after KTX), delayed graft function (DGF, defined as any need for dialysis within the first week after KTX), biopsy-proven acute rejection, new onset of diabetes after transplantation, and the following cardiovascular events: myocardial infarction, angina pectoris, coronary artery revascularization, or congestive heart failure after transplantation.

### Statistical Analysis

Group comparisons were performed using the Mann-Whitney U test for not normally distributed data, Fisher’s exact test for categorical variables, and the Student’s t-test for normally distributed data. Normally distributed continuous variables (tested by the Kolmogorov-Smirnov test) were shown as mean with standard deviation (SD), and not normally distributed continuous variables were presented as median with interquartile range (IQR). The probability of event-free survival, which included patient survival and the probability of graft loss, was estimated using the Kaplan-Meier method, and all three endpoints were compared using the log-rank test (for *p*-values ≤ 0.05). Recipient kidney function (eGFR) was analyzed using a mixed model for repeated measurements. Time points in each group were compared using a one-way analysis of variance (ANOVA). Additionally, the DS+ group was compared to the DS− group within each time point. All *p*-values were adjusted using the Holm-Šídák method. Results are presented as the median and a 95% confidence interval. Cox proportional hazards regression models were fitted to determine the influence of donor variables (smoking, age, cold ischemia time, warm ischemia time, CPR, sCr at procurement, hypertension, diabetes mellitus, ECD and KDPI) on event-free survival, patient survival, graft loss, as well as reduced renal function (transformed to a dichotomous endpoint of eGFR </> 30 mL/h/1.73 kg^2^). To solely focus on donor variables, recipient characteristics were omitted in the Cox proportional hazards regression models. Hazard ratios (HR) and 95% confidence intervals (CI) were calculated. All statistical analyses and graphics were performed using IBM SPSS^®^ Statistics 24 for Windows (IBM Corporation, Somers, NY, United States) and GraphPad Prism 10 software for Windows (GraphPad Software, CA, United States).

## Results

Five hundred and eighty patients were found eligible and were further stratified based on the history of smoking in the deceased donor. Within the study’s cohort, 276 patients (47.6%) received a graft from a smoking donor, and 304 patients (52.4%) received a graft from a non-smoking donor (Figure 1).

With respect to demographic parameters, kidney donors in the DS+ and DS− cohorts were largely comparable (Table 1). However, the DS− group was older (56.8 vs. 51.1 years; *p* < 0.001), included more ECD donors (*n* = 174 [57.2%] vs. *n* = 115 [41.7%]; *p* < 0.001), and showed a less favorable HLA mismatch (Table 1). Baseline recipient demographics were also largely comparable (Table 2). However, recipients in the DS− cohort were significantly more often diagnosed with chronic pyelonephritis compared to the DS+ group.

**TABLE 1:** Donor characteristics.

	DS– *n* = 304	DS+ *n* = 276	*p*-value
Age (years, mean ± SD)	56.79 ± 16.23	51.11 ± 12.04	**<0.001** ^a^
Sex (n, % males)	143 (47.0)	144 (52.2)	0.217^b^
Body mass index [kg/m^2^, median (IQR)]	26.0 (24.0; 28.0)	26.0 (24.0; 29.0)	0.738^c^
Cardiopulmonary resuscitation (n, %)	58 (19.1)	69 (25.0)	0.085^b^
Duration of cardiac arrest [min, median (IQR)]	20.00 (10.00; 46.25)	20.00 (10.00; 40.00)	0.401^c^
Hypertension (n, %)	98 (32.2)	88 (31.9)	0.928^b^
Diabetes mellitus (n, %)	32 (10.5)	21 (7.6)	0.223^b^
Cold ischemia time [h, median, (IQR)]	10.03 (7.19; 13.40)	11.00 (8.09; 13.40)	0.126^c^
Kidney donor profile index [median, (IQR)]	67.00 (46.00; 87.00)	69.50 (46.00; 91.00)	0.566^c^
Kidney donor risk index [median, (IQR)]	1.20 (0.97; 1.54)	1.21 (0.98; 1.63)	0.583^c^
Extended criteria donors (n, %)	174 (57.2)	115 (41.7)	**<0.001** ^b^
Perioperative vasopressors (n, %)	44 (14.5)	30 (10.9)	0.194^b^
Time at intensive care unit prior to donation [days, median, (IQR)]	3.0 (2.0; 6.0)	3.0 (2.0; 7.0)	0.821^c^
Diuresis prior to donation [m/h, median (IQR)]	160.0 (108.3; 221.6)	159.9 (100.0; 229.9)	0.752^c^
Cytomegalovirus risk status			0.518^b^
low (n, %)	98 (32.2)	100 (36.2)	
Intermediate (n, %)	79 (26.0)	63 (22.8)	
High (n, %)	127 (41.8)	112 (40.6)	
Human leukocyte antigen mismatch		
0 (n, %)	49 (16.1)	54 (19.6)	**0.017** ^b^
1–3 (n, %)	151 (49.7)	157 (56.9)	
4–6 (n, %)	103 (33.9)	64 (23.2)	
Multiple renal arteries (>1)			
(n, %)	59 (19.4)	62 (22.6)	0.454^b^

Results are presented as mean ± standard deviation (SD), median with interquartile range (IQR), or relative frequency. Cytomegalovirus risk status based on donor (d) and recipient (r) status: low = d−/r−, intermediate = d−/r+ or d+/r+, high = d+/r−.

^a^ Student’s t-test.

^b^ Chi-square test.

^c^ Mann-Whitney U test. Significant p values are highlighted in bold for clarity.

**TABLE 2:** Recipient characteristics.

	DS– *n* = 304	DS+ *n* = 276	*p*-value
Age (mean ± SD)	57.78 ± 12.94	54.05 ± 11.71	0.171^a^
Sex (n, % male)	188 (61.8)	171 (62.0)	0.977^b^
Dialysis vintage [months, median, (IQR)]	58.0 (33.0; 88.0)	78.0 (48.0; 99.75)	0.199^c^
Hypertension before transplantation (*n*, %)	267 (87.8)	246 (89.1)	0.624^b^
Diagnosis of end-stage renal disease (*n*, %)			
Glomerulonephritis	101 (33.2)	99 (35.9)	0.541^d^
Diabetic nephropathy	23 (7.6)	28 (10.1)	0.306^d^
Hypertensive nephropathy	21 (6.9)	18 (6,5)	0.870^d^
Obstructive nephropathy	3 (1.0)	2 (0.7)	>0.999^d^
Fokal segmental glomerulosklerosis	14 (4.6)	13 (4.7)	>0.999^d^
Interstitial nephritis	9 (3.0)	17 (6.2)	0.072^d^
Vasculitis	6 (2.0)	4 (1.4)	0.755^d^
Chronic pyelonephritis	18 (5.9)	4 (1.4)	**0.005** ^d^
Alport Syndrome	3 (1.0)	6 (2.2)	0.321^d^
Autosomal dominant polycystic kidney disease 2	38 (12.5)	35 (12.7)	>0.999^d^
Benign nephrosclerosis	5 (1.6)	6 (2.2)	0.764^d^
Other	62 (20.4)	42 (15.2)	0.129^d^

Results are presented as mean ± standard deviation (SD), median with interquartile range (IQR), or relative frequency.

^a^ Student’s t-test.

^b^ Chi-square test.

^c^ Mann-Whitney U test.

^d^ Fischer’s exact test. Significant *p* values are highlighted in bold for clarity.

**FIGURE 2:**
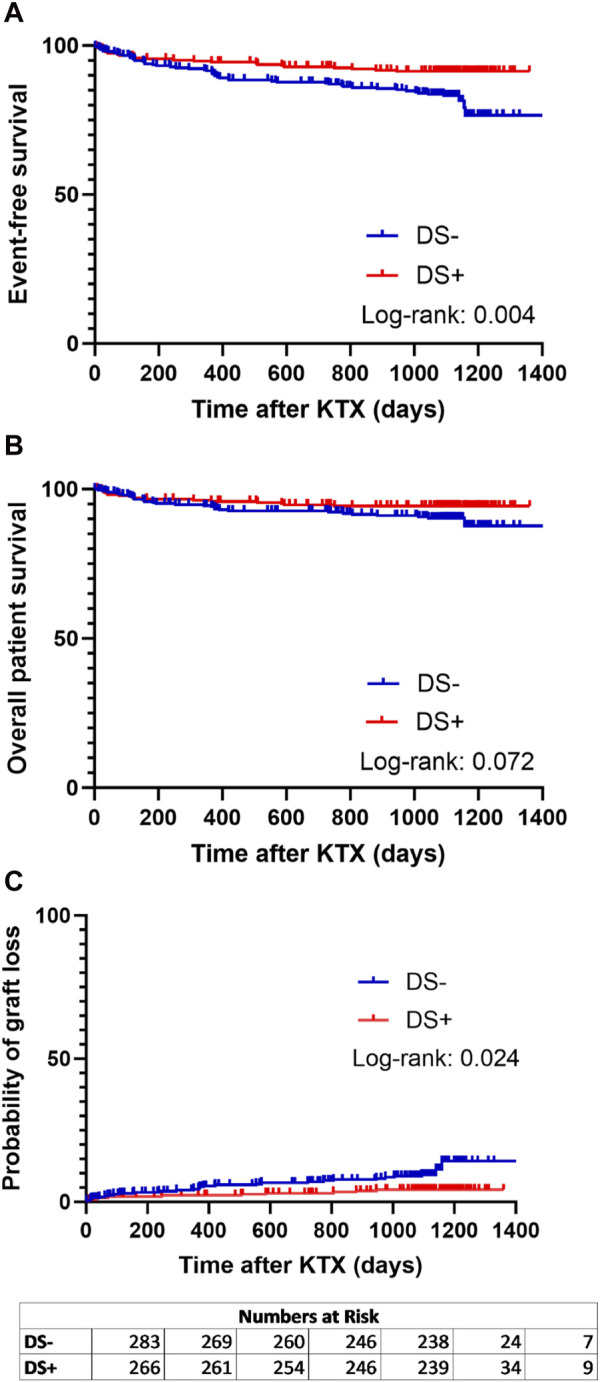
Analysis of event-free survival **(A)** (defined as combined patient and graft survival), **(B)** overall patient survival, and **(C)** probability of graft loss separated for patients receiving a graft from a smoking donor (DS+) and patients receiving a graft from a non-smoking donor (DS−). Survival rates of DS+ (red lines) and DS− (blue lines) recipients following kidney transplantation (KTX) were estimated using Kaplan-Meier methodology and compared using the log-rank test.

When analyzing the primary endpoint, a higher probability of event-free survival (combined patient and graft survival) was observed in the DS+ group compared to the DS− group (*p* = 0.004) (Figure 2A). Interestingly, long-term patient survival did not differ significantly between both groups (*p* = 0.072) (Figure 2B). Nevertheless, the probability of graft loss was higher in patients who received a DS− graft than in those who received a DS+ graft (*p* = 0.024) (Figure 2C).

**FIGURE 3:**
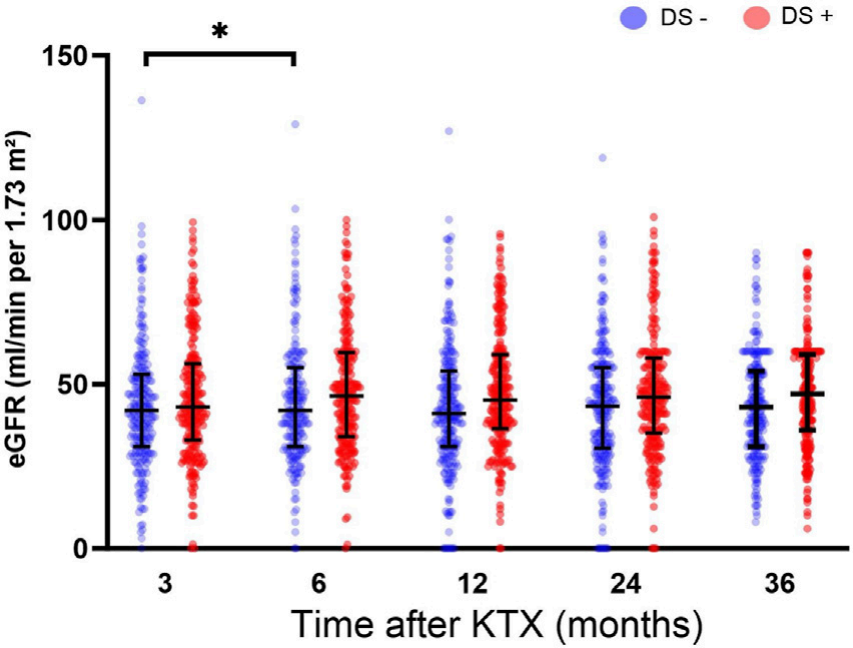
Estimated glomerular filtration rate (eGFR mL/min/1.73 m^2^) for analysis of post-transplant graft function up to 36 months after KTX. Comparisons of eGFR within each group were conducted using a one-way analysis of variance (ANOVA). The DS+ group was compared to the DS− group within each time point. All *p*-values were adjusted using the Holm-Šídák method. A *p*-value less than 0.05 was considered statistically, *p ≤ 0.05; **p ≤ 0.01; ***p ≤ 0.001; ****p ≤ 0.0001.

The DS+ and the DS− cohorts exhibited comparable renal function at 3, 6, 12, 24, and 36 months after KTX (Figure 3). However, significantly higher eGFR rates were observed in the DS− cohort at 6 months after KTX compared to the 3-month baseline (*p* = 0.022). Similarly, renal function after KTX, estimated by PE and UPCR at 1, 2 and 3 years after KTX, demonstrated comparable results for the DS+ and the DS− groups (Table 3). Comparison of the additional secondary endpoints showed no differences between the DS+ and DS− cohorts in the incidence of DGF, PNF, biopsy-proven rejection, new-onset of diabetes after transplantation, or cardiovascular events after KTX (Table 3).

**TABLE 3:** Secondary endpoints.

	DS− *n* = 304	DS+ *n* = 276	*p*-value
Primary non-function (*n*, %)	11 (3.6)	18 (6.5)	0.089^a^
Delayed graft function (*n*, %)	65 (21.4)	69 (25.0)	0.330^a^
Biopsy-proven acute rejection (*n*, %)	152 (50.0)	129 (46.7)	0.259^a^
New onset of diabetes after transplantation (*n*, %)	40 (13.2)	33 (12.0)	0.176^a^
Cardiovascular event after transplantation (*n*, %)	30 (9.9)	27 (9.8)	0.972^a^
Parameters of kidney function (mean ± SD)			
Protein excretion per day	13.41 ± 23.00	14.73 ± 40.04	0.417^b^
1 year after KTX (mg/d)			
Protein excretion per day	13.63 ± 26.56	10.90 ± 18.47	0.243^b^
2 years after KTX (mg/d)			
Protein excretion per day	16.59 ± 41.78	15.71 ± 39.82	0.972^b^
3 years after KTX (mg/d)			
Urine protein/creatinine ratio	210.6 ± 358.5	236.2 ± 704.6	0.988^b^
1 year after KTX (mg/g creatinine)			
Urine protein/creatinine ratio	213.1 ± 419.1	178.7 ± 409.5	0.453^b^
2 years after KTX (mg/g creatinine)			
Urine protein/creatinine ratio	224.2 ± 547.5	232.1 ± 626.2	0.928^b^
3 years after KTX (mg/g creatinine)			

Results are presented as mean ± standard deviation (SD), median with interquartile range (IQR), or relative frequency.

^a^ Chi-square test.

^b^ Mixed effects model, *p*-values were adjusted using the Holm-Šídák method.

**FIGURE 4:**
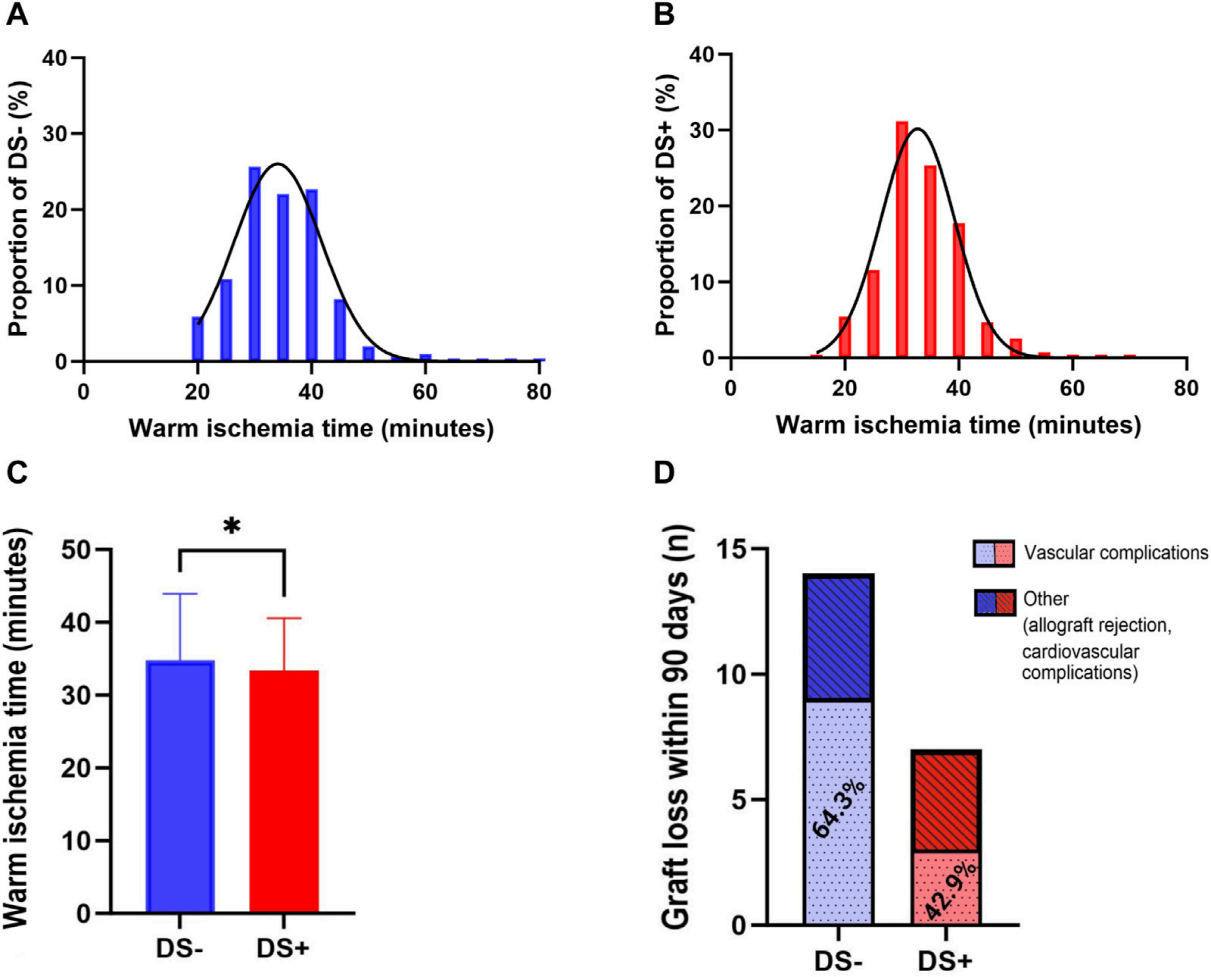
Distribution of warm ischemia time (WIT) within the DS+ and DS− cohorts by histogram **(A, B)**. Direct comparison of the DS− and DS+ cohorts by Mann Whitney U. All *p*-values were adjusted using the Holm-Šídák method **(C)**. Plotting of significant values (*p = 0.024). Proportions of complications leading to graft loss within 90 days after KTX **(D)**, including vascular complications and others, specified as allograft rejection or cardiovascular complications. Direct comparison of the DS+ and DS− cohorts (red and blue bars) by Fisher’s exact test.

Since DS is thought to be associated with the development of macroscopic renal artery arteriosclerosis, implantation times were analyzed for the DS− (Figure 4A) and DS+ (Figure 4B) cohorts. Figure 4C illustrates that WIT was longer in the DS− cohort (35.0 min vs. 33.5 min; *p* = 0.047). Additionally, donor arteriosclerosis might pose a technical challenge when conducting arterial anastomosis, subsequently resulting in technical and thrombotic vascular complications. Therefore, the proportion of vascular complications (including postoperative bleeding and vascular occlusion) was further analyzed (Figure 4D). Overall, a low rate of vascular complications leading to graft loss within 90 days was present in both cohorts. Interestingly, the relative number of graft losses due to vascular complications was higher in the DS− group (64.3%) compared to the DS+ cohort (42.9%); however, the comparison was not noticeable (*p* = 0.397).

Univariate and multivariate Cox regression models were used to analyze independent donor-associated risk factors. The following endpoints were explored: event-free survival (including patient and graft survival), patient survival, graft survival, and marginal renal function (eGFR <30 mL/h/1.73 m^2^, Tables 4–7). As presented in Table 4, the Cox regression model revealed that DS status was associated with event-free survival in the univariate analysis (HR [0.48; 0.29–0.80], *p* = 0.005), but did not reach statistical significance in multivariate analysis (HR [0.62; 0.35–1.09], *p* = 0.095). Similar, DS positively affected the probability of graft loss in the univariate analysis (HR [0.43; 0.21–0.86], *p* = 0.017), but did not reach statistical significance in multivariate analysis (Table 5). DS status was not associated with patient survival (Table 6). Furthermore, regarding renal function, DS+ status was associated with better (eGFR >30 mL/h/1.73 m^2^) graft function in the univariate analysis (HR [0.55; 0.38–0.80], *p* = 0.002), but did not reach statistical significance in multivariate analysis (Table 7).

**TABLE 4:** Cox regression model of event-free survival.

Donor characteristics	Univariate		Multivariate	
	*p*-value	HR (95% Cl)	*p*-value	HR (95% Cl)
Smoking (yes/no)	**0.005**	0.48 (0.29–0.80)	0.095	0.62 (0.35–1.09)
Age (years)	**<0.001**	1.03 (1.01–1.05)	**0.003**	1.03 (1.01–1.05)
Cold ischemia time (hours)	0.925	1.00 (0.94–1.06)	0.171	1.04 (0.98–1.11)
Warm ischemia time (min)	0.236	1.02 (1.00–1.04)	0.475	1.01 (0.98–1.04)
Cardiopulmonary resuscitation (yes/no)	0.681	0.88 (0.49–1.59)	0.963	1.015 (0.54–1.90)
Serum creatinine (µmol/L)	0.989	1.00 (1.00–1.00)	0.146	1.00 (1.00–1.00)
Hypertension (yes/no)	0.083	1.52 (0.95–2.45)	0.333	1.30 (0.77–2.19)
Diabetes mellitus (yes/no)	0.782	1.12 (0.51–2.44)	0.776	0.89 (0.39–2.03)
Kidney donor risk index	0.456	1.00 (0.99–1.01)	0.140	0.99 (0.98–1.00)
Extended criteria donor (yes/no)	**0.025**	1.83 (1.08–3.10)	**0.019**	1.96 (1.12–3.43)

HR, Hazard ratios; CI, 95% confidence intervals.

Significant *p* values are highlighted in bold for clarity.

**TABLE 5:** Cox regression model of graft loss.

Donor Characteristics	Univariate		Multivariate	
	*p*-value	HR (95% Cl)	*p*-value	HR (95% Cl)
Smoking (yes/no)	**0.017**	0.43 (0.21–0.86)	0.102	0.51 (0.23–1.14)
Age (years)	**0.001**	1.04 (1.02–1.07)	**0.009**	1.04 (1.01–1.07)
Cold ischemia time (hours)	0.434	0.97 (0.89–1.05)	0.927	1.00 (0.92–1.08)
Warm ischemia time (min)	0.170	1.02 (0.99–1.06)	0.424	1.02 (0.98–1.05)
Cardiopulmonary resuscitation (yes/no)	0.797	1.10 (0.52–2.33)	0.325	1.50 (0.67–3.36)
Serum creatinine (µmol/L)	0.774	1.00 (0.99–1.00)	0.917	1.00 (0.99–1.01)
Hypertension (yes/no)	**0.018**	2.16 (1.14–4.08)	0.253	1.50 (0.75–3.02)
Diabetes mellitus (yes/no)	0.381	1.52 (0.59–3.90)	0.944	1.04 (0.38–2.85)
Kidney donor risk index	0.193	0.99 (0.98–1.00)	0.078	0.99 (0.98–1.00)
Extended criteria donor (yes/no)	0.193	1.59 (0.79–3.20)	0.138	1.76 (0.83–3.69)

HR, Hazard ratios; CI, 95% confidence intervals.

Significant *p* values are highlighted in bold for clarity.

**TABLE 6:** Cox regression model of patient survival.

Donor characteristics	Univariate		Multivariate	
	*p*-value	HR (95% Cl)	*p*-value	HR (95% Cl)
Smoking (yes/no)	0.093	0.57 (0.30–1.10)	0.731	0.88 (0.43–1.81)
Age (years)	**0.008**	1.03 (1.01–1.10)	**0.025**	1.03 (1.00–1.06)
Cold ischemia time (hours)	0.877	0.99 (0.92–1.07)	0.169	1.06 (0.98–1.14)
Warm ischemia time (min)	**0.024**	1.04 (1.01–1.07)	**0.032**	1.03 (1.00–1.06)
Cardiopulmonary resuscitation (yes/no)	0.480	0.75 (0.33–1.69)	0.559	0.77 (0.31–1.88)
Serum creatinine (µmol/L)	0.985	1.00 (1.00–1.00)	0.110	1.00 (1.00–1.01)
Hypertension (yes/no)	0.264	1.43 (0.76–2.70)	0.386	1.36 (0.68–2.70)
Diabetes mellitus (yes/no)	0.833	1.12 (0.34–3.14)	0.854	0.90 (0.30–2.69)
Kidney donor risk index	0.963	1.00 (0.99–1.01)	0.416	0.99 (0.98–1.01)
Extended criteria donor (yes/no)	**0.030**	2.24 (1.08–4.63)	**0.027**	2.38 (1.10–5.13)

HR, Hazard ratios; CI, 95% confidence intervals.

Significant *p* values are highlighted in bold for clarity.

**TABLE 7:** Cox regression model of renal function.

Donor characteristics	Univariate		Multivariate	
	*p*-value	HR (95% Cl)	*p*-value	HR (95% Cl)
Smoking (yes/no)	**0.002**	0.55 (0.38–0.80)	0.495	0.86 (0.56–1.33)
Age (years)	**<0.001**	1.05 (1.03–1.06)	**<0.001**	1.04 (1.02–1.06)
Cold ischemia time (hours)	**0.043**	0.95 (0.91–1.00)	0.710	1.01 (0.96–1.07)
Warm ischemia time (min)	0.102	1.02 (1.00–1.04)	0.070	1.02 (1.00–1.05)
Cardiopulmonary resuscitation (yes/no)	0.157	0.70 (0.42–1.15)	0.483	0.82 (0.47–1.44)
Serum creatinine (µmol/L)	0.624	1.00 (1.00–1.00)	0.617	1.00 (1.00–1.00)
Hypertension (yes/no)	**0.002**	1.77 (1.23–2.55)	0.077	1.45 (0.96–2.19)
Diabetes mellitus (yes/no)	**0.011**	1.89 (1.16–3.10)	0.845	0.94 (0.51–1.74)
Kidney donor risk index	0.817	1.00 (1.00–1.01)	0.308	0.99 (0.99–1.00)
Extended criteria donor (yes/no)	0.343	1.21 (0.82–1.80)	0.337	1.23 (0.81–1.88)

HR, Hazard ratios; CI, 95% confidence intervals.

Significant *p* values are highlighted in bold for clarity.

Donor age (univariate analysis (HR [1.03; 1.01–1.05], *p* < 0.001), multivariate analysis (HR [1.03; 1.01–1.05], *p* = 0.003)) and ECD status (univariate analysis (HR [1.83; 1.08–3.10], *p* = 0.025), multi-variate analysis (HR [1.96; 1.12–3.43], *p* = 0.019)) were significantly associated with worse event-free survival in the Coy regression analyses (Table 4). Similarly, donor age contributed to a higher probability of graft loss in univariate (HR [1.04; 1.02–1.07], *p* = 0.001) and multivariate (HR [1.04; 1.01–1.07], *p* = 0.009) Cox regression models (Table 5). Patient survival (Table 6) was also negatively influenced by donor age (univariate analysis (HR [1.03; 1.01–1.10], *p* = 0.008), multivariate analysis (HR [1.03; 1.00–1.06], *p* = 0.025)) and ECD status (univariate analysis (HR [2.24; 1.08–4.63], *p* = 0.030), multi-variate analysis (HR [2.38; 1.10–5.13], *p* = 0.027)). Finally, donor age was associated with impaired renal function in univariate (HR [1.05; 1.03–1.06], *p* < 0.001) and multivariate (HR [1.04; 1.02–1.06], *p* < 0.001) analyses (Table 7).

## Discussion

The shortage of donor organs for kidney transplantation is undoubtedly a pressing issue for the transplant community. Additionally, demographic changes in society and increasingly poor donor quality are leading to a more and more demanding kidney allocation process in which donor-associated characteristics must be critically balanced. Therefore, the aim of this study was to investigate the role of cigarette smoking as a potential donor-associated risk factor and its long-term effects after KTX in a representative and contemporary cohort from the ET area. Over a 36-month follow-up period, this study evaluated 580 patients for survival (patient and graft) and functional outcomes after receiving a kidney allograft from a smoking or non-smoking deceased donor. Overall, this study found no evidence of inferiority of grafts from cigarette-smoking deceased donors. In addition, this study found no affirmation of an increased risk for recipients. In contrast, we observed that the primary composite endpoint of event-free survival and graft survival was better in the DS+ cohort when analyzed using the Kaplan-Meier method.

One approach to explain the findings in the DS+ group might be the analysis of the DS− baseline characteristics. The DS− cohort had a higher WIT, older age, poorer HLA matching and a higher ECD rate, which may have negatively affected patient and graft survival. In line with this, the postoperative increase in eGFR 6 months after KTX compared to baseline in DS− patients could indicate impaired kidney allograft function in the DS− cohort.

The positive results for the DS+ cohort should not be interpreted in favor of a positive donor smoking history in KTX. Moreover, this demonstrates an inherent and rather worrying bias. One could argue that DS is currently perceived as an additional risk factor, and a smoking history might encourage transplant professionals to decline an offered kidney graft, for which smoking is the tipping point. Hence, one could suggest that if transplant professionals accept a kidney graft from a donor with a history of smoking, other donor-associated factors (e.g., age or HLA matching) must be in favor of using that graft. Accordingly, the observed results of DS as a protective factor associated with improved graft survival should be interpreted with caution, not because of the misinterpretation that DS is protective (for which no logical pathophysiological explanation can be found), but rather because it reflects the direct impact of DS on allocation. We hypothesize that many suitable organs from smoking donors must have been rejected to create such a favorable outcome, as demonstrated in this analysis. Therefore, DS might represent a potentially misleading selection bias in kidney allograft allocating, which is a dilemma in today’s era of donor organ shortage and decreasing organ quality, especially, since there is no substantial evidence that DS adversely affects long-term patient or allograft outcomes. Thus, cigarette smoking should not be used as a reason to accept a potentially less suitable donor. More importantly, however, DS should not be considered as a deciding factor in refusing a kidney graft.

Although it appears highly unlikely that donor smoking has a direct, causative positive effect on the outcome in our study cohort it is noteworthy that smoking has been found to be protective in other disease. A “smoker’s paradox,” referring to the decreased mortality in smokers after acute coronary syndrome and stroke, has been described, but the available data is limited, partially questionable and has been refuted by more recent analyses [18, 19]. Nevertheless, there is robust evidence for a protective effect of smoking on the risk of Parkinson’s disease [20] and ulcerative colitis [21]. However, plausible biologic mechanisms remain scare. One possible explanation is the immunomodulatory and anti-inflammatory effect of nicotine mediated by the activation of nicotinic acetylcholine receptor α7 in immune cells, but it remains questionable if this donor-associated protective mechanism can translate into long-term improvement in the recipient and outweigh the proven negative effects of smoking.

Only a few studies have evaluated smoking as a donor-associated risk factor in KTX, and there is an ambiguity in the current literature. Heldt et al. and Underwood et al. conducted single-center studies to investigate DS in living kidney donation and reported variable results. On the one hand, Heldt et al. showed a significantly lower graft function (GFR) in recipients from smoking donors, whereas Underwood et al. did not demonstrate an effect of DS on graft survival, but observed a negative correlation between DS and recipient survival [12, 13]. Two studies have focused on the effect of DS in deceased donation. Lin et al. demonstrated that DS was associated with an increased risk of graft loss (adjusted HR = 1.05, *p* = 0.028) and impaired patient survival (adjusted HR = 1.06, *p* = 0.021) in a retrospective registry analysis (United Network for Organ Sharing dataset) of deceased donors, including non-heart-beating donors, between 1994 and 1999 [10]. Later, Gillott et al. carried out a registry analysis (United Kingdom Transplant Registry, including patients from 2001 to 2013) and confirmed increased recipient mortality in a cohort receiving DS grafts (HR = 1.12, *p* = 0.044). However, no effect on graft survival was observed [11]. Thus, DS might affect patient-related outcomes more frequently than kidney allograft function and consequently graft survival. Gillott et al. revised possible approaches to explain impaired patient-related outcomes after receiving a kidney allograft from a smoking donor. The authors argue that the association between smoking and endothelial dysfunction might have a synergistic effect with other recipient comorbidities, which could increase mortality. Another possible explanation could be immune-related alterations and interactions that could be associated with increased mortality. Nevertheless, evidence of long-term pathophysiological consequences of DS leading to impaired patient survival remains scarce.

It is paramount to critically compare the above findings with our data. First, our study is the first in the field of ET. This is important because there are well-described and profound differences in demographics, allocation, and patient and graft survival outcomes between the United States, the United Kingdom, and the ET region [22]. Thus, direct comparisons remain difficult. Second, these findings may also indicate a changing role of DS in KTX over time, particularly in the face of donor shortages and increasing rates of ECD. In line with this, when only ECD from the United Network for Organ Sharing dataset was analyzed, no negative effect of DS on graft survival was found [23].

Nevertheless, our data set and analysis have several limitations. First, we do not have adequate information regarding the respective pack years for the DS+ cohort, which would have allowed us to perform a much more granular analysis, calculate a linear relationship, and conduct subgroup analysis stratified by pack years. This is of special interest since smoking-associated histological injury and graft function after KTX depend on the donor’s cumulative smoking dose [13, 15]. However, lacking this information adds a more realistic and real-world aspect to our study, as it represents the actual information on which the transplant professionals involved have to base their decision on whether to decline or accept a kidney offer. In addition, there is no official data regarding the smoking prevalence among organ donors within the ET area. Therefore, one could only gauge the possible impact of discarding organs from smokers on the current organ shortage. However, the age-standardized prevalence of smoking among individuals aged 15 years and older in Western Europe is between 22.7% (female) and 28.8% (male) [6]. Since approximately 97% of all kidney donors in the ET area are 15 years and older, this further illustrates the impact of discarding otherwise suitable kidney grafts based on DS. Moreover, our findings need to be evaluated concerning the sample size, which represents an additional limitation of this retrospective cohort study. On the other hand, the recipient cohort included can be regarded as advantageous for this investigation since baseline characteristics or immunosuppression protocols show no differences. As it has been previously argued that DS exerts its potentially negative effects via the development of glomerulosclerosis in the donor, the availability of implantation biopsies would have also strengthened the study. Unfortunately, our data set has a very low frequency of biopsies, which does not allow further analysis.

## Conclusion

This retrospective cohort study investigated 580 patients regarding the effect of DS on graft longevity and recipient survival with a 36-month follow-up. We observed a significant improvement in the primary composite endpoint, including patient survival and the probability of graft loss, in the DS+ cohort. However, this favorable effect of DS+ was not noticeable after controlling for other donor-associated factors using multivariate analysis. Thus, this study found no evidence of inferiority of grafts from cigarette-smoking deceased donors and no evidence of an increased risk for recipients. In conclusion, we strongly suggest caution in declining kidney allografts that are potentially suitable but do have a positive cigarette smoking status.

## Data Availability

The data analyzed in this study is subject to the following licenses/restrictions: The raw data supporting the conclusion of this article will be made available by the authors upon reasonable request. Requests to access these datasets should be directed to felix.becker@ukmuenster.de.
